# Clinicoradiological Profile of COVID-19–Associated Rhino-Orbital Cerebral Mucormycosis with a Focus on Computed Tomography: A Clinical Case Series and Review

**DOI:** 10.4269/ajtmh.22-0298

**Published:** 2023-07-24

**Authors:** Anuj Prabhakar, Sandeep Bansal, Sameer Vyas, Vikas Bhatia, Ajay Kumar, Sourabha Patro, Usha Singh, Naresh Panda, Paramjeet Singh, Ashish Bhalla, Goverdhan Dutt Puri, Chirag Kamal Ahuja

**Affiliations:** ^1^ Division of Neuroimaging & Interventional Neuroradiology, Department of Radiodiagnosis, Postgraduate Institute of Medical Education and Research, Chandigarh, India;; ^2^ Department of Otolaryngology-Head and Neck Surgery, Postgraduate Institute of Medical Education and Research, Chandigarh, India;; ^3^ Department of Ophthalmology, Postgraduate Institute of Medical Education and Research, Chandigarh, India;; ^4^ Department of Internal Medicine, Postgraduate Institute of Medical Education and Research, Chandigarh, India;; ^5^ Department of Anaesthesia, Postgraduate Institute of Medical Education and Research, Chandigarh, India

## Abstract

COVID-19–associated rhino-orbital cerebral mucormycosis (ROCM) has a rapidly evolving course with high morbidity and mortality. We describe imaging features of COVID-19–associated ROCM based on noncontrast computed tomography (NCCT). This retrospective single-center observational study included 50 patients with COVID-19 from January 1, 2021 to June 30, 2021 who subsequently developed ROCM confirmed by fungal culture studies. All patients underwent NCCT of the paranasal sinuses as the diagnostic workup. The involvement of the nasal cavity, paranasal sinuses, orbits, and intracranial cavity was identified and graded. The ethmoid sinuses were most commonly involved [right (*n* = 46 of 50) > left (*n* = 45 of 50)], followed by the maxillary, sphenoid, and frontal sinuses. Thinning and erosions of the hard palate were noted in 18% of patients (*n* = 9), whereas 34% (*n* = 17) showed dehiscence of the lamina papyracea. Retromaxillary fat stranding was noted in 68% of patients (*n* = 34). Severe ethmoid sinusitis was associated significantly with ipsilateral pterygopalatine fossa involvement. The extraocular muscles were involved in 64% of patients (*n* = 32), with 84% (*n* = 42) showing orbital fat stranding. Proptosis of the affected eye was seen in 66% of patients, optic nerve involvement in 52%, and irregularity of globe contour in 12% (*n* = 6). The cavernous sinuses were affected in 10% of patients (*n* = 5), with three of them having temporal infarcts. COVID-19–associated ROCM is an acute, invasive fungal disease characterized by multisinus involvement, often with orbital and intracranial extension. Bilateral involvement with rapid progression should alert one to underlying COVID-19 disease.

## INTRODUCTION

The COVID-19 pandemic that began in 2019 has been associated with a variety of opportunistic infections. *Candida* and *Aspergillus* have been identified as the most common opportunistic fungal infections in patients with COVID-19.^1^ However, an increasing number of mucormycosis coinfections in both active and recovering COVID-19 cases has been reported globally. The prime cause of this surge may be an ideal milieu provided by hypoxia, hyperglycemia, steroid intake, metabolic acidosis, diabetic ketoacidosis, elevated ferritin, and immunosuppression, along with other risk factors. Uncontrolled diabetes is considered a significantly critical risk factor.^2^ In addition, it has been proposed that there may be molecular associations between COVID-19 and mucormycosis. The concentration of the molecule GRP78, a heat-shock protein, is increased in COVID-19 infection and has increased binding capacity to *Rhizopus* germlings, leading to increased invasive capability of Mucorales infection.^3^

Mucormycosis is a potentially life-threatening fungal infection caused by zygomycetes.^4^ These fungi have been cultured from air and soil samples.^5^^,^^6^ The taxonomic classification of Mucorales includes 55 genera and 260 species.^7^ Species commonly found in India include *Rhizopus*, *Cunninghamella*, *Lichtheimia*, *Apophysomyces*, and *Rhizomucor*.^8^ *Rhizopus arrhizus* is the most commonly isolated fungi in patients with mucormycosis from India. Mucorales species usually afflict patients who have suboptimal immunity. Clinically, the presentation varies by the affected anatomic site. The commonly involved sites include pulmonary, rhino-orbital cerebral, gastrointestinal, renal, and cutaneous forms or a combination thereof.^5^ The rhino-orbital cerebral type of mucormycosis is most common in India, followed by pulmonary and cutaneous mucormycosis^4^. Rhino-orbital cerebral mucormycosis (ROCM) has an overall survival rate of only 60%.^9^ Thus, adequate and timely intervention is an important factor in the management of this disease.

With timely intervention being the key in the management of mucormycosis, it is significant that an adequate preoperative workup be done for patients before going ahead with the criteria standard management of debridement and subsequent antifungal therapy. Clinical examination in cases of mucormycosis is grossly inadequate to make surgical plans, because the sites of affection are not visible during routine clinical examinations. Hence, radiological investigations such as computed tomography (CT) and magnetic resonance imaging (MRI) do play a significant role in determining the extent of disease and the surgical debridement required for case management.

Magnetic resonance imaging is not widely available and is time-consuming, and accomplishing it in patients with suspected or proven COVID-19 while maintaining all barrier precautions (when many patients are using oxygen supplementation) is impractical. Hence, and especially during the pandemic, we resorted to using CT scans to identify the extent of disease and surgery required, because it is easy to conduct and has widespread availability, short examination times (leading to less chances of cross-infection), and simpler sanitization methods compared with MRI.

In this series, we compile, analyze, and discuss the CT scans and clinical features of patients with COVID-19 who also developed ROCM.

## MATERIALS AND METHODS

### Subjects.

Of the 58 consecutive patients who presented with clinical suspicion of rhino-orbital fungal infection during a 6-month period, 50 were included in our retrospective, single-center observational study. The enrolled patients were positive for COVID-19 as confirmed by reverse transcription–polymerase chain reaction (RT-PCR) who developed ROCM subsequently, which was confirmed by fungal culture studies. Patients for whom > 14 days had passed since the diagnosis were considered “post-COVID-19.” The following inclusion criteria needed to be met for enrollment: 1) patients older 18 years, 2) a positive COVID-19 RT-PCR test during the past 3 months from current presentation of rhino-orbital mucormycosis, and 3) any symptom suggestive of paranasal sinus, orbital or cavernous sinus, or brain involvement, which indicated a clinical fungal infection. Exclusion criteria included 1) patients with non-COVID-19–associated mucormycosis, 2) patients who refused endoscopic surgery, 3) patients who were discharged but in whom clinical (in-person/telephone) follow-up was not available, and 4) patients who did not give consent for enrollment in the study.

All patients meeting our inclusion criteria were admitted to a dedicated facility for COVID-19 management. The facility, the Postgraduate Institute of Medical Education and Research (Chandigarh, India), is a 2,400-bed tertiary care public hospital and research center in northern India that designated 250 beds for managing patients with COVID-19 from its territory and adjoining states during the peak of the pandemic.

### Imaging protocol.

All patients underwent noncontrast computed tomography (NCCT) (Somatom Definition AS, 128-slice Siemens medical system, Erlangen, Germany) for the paranasal sinuses as a part of the diagnostic workup. After confirming that the quality of acquired images was appropriate diagnostically, two neuroradiologists with 9 (C. K. A.) and 25 (P. S.) years of experience reviewed the imaging findings. Both were blinded to the clinical information and endoscopy findings, as well as the future management plan. A consensus was reached in case of discrepancies regarding abnormalities.

### Image characterization.

The involvement of the paranasal sinuses was described as mild if there was minimal mucosal thickening, moderate if < 50% of the sinus was opacified, and severe if > 50% of the sinus was opacified (as agreed by the authors). Focal thinning, erosions, or a breach in continuity suggested the involvement of adjacent bones. Fat stranding, loss of normal fat planes, and ill-defined soft tissue thickening were looked for to assess the extent of disease into the surrounding soft tissue. Orbital involvement was evaluated based on the presence or absence of fat stranding or soft tissue density in the orbital fat, and an increase in the bulk of the extraocular muscles with or without surrounding stranding. Increase in the bulk of the optic nerve, irregularity of nerve sheath margins, and significant perisheath fat stranding on NCCT were considered indicators of optic nerve involvement. Intracranial spread was defined by the involvement of the cavernous sinuses or parenchymal infarcts. Bulky cavernous sinuses with an increase in the convexity of the outer margin were considered to be indicators of cavernous sinus involvement. Hypodensities within the adjacent temporal lobe or the visualized brain parenchyma were considered signs of parenchymal involvement.

### Statistical analysis.

Statistical analysis was performed using IBM SPSS Statistics for Windows (Version 26.0. Armonk, NY). Descriptive statistics for qualitative data—namely, age, gender, sinus involvement, bony destruction, and orbital and intracranial involvement—are presented as frequencies and percentages. Associations between variables were explored using the χ^2^ test and unpaired *t*-test for qualitative and quantitative variables, respectively.

## RESULTS

### Demographics.

The average age of our cohort was 54 years. There were 13 women and 37 men. Twenty-three of 50 patients (46%) were post-COVID-19. The remaining 54% were in the active phase of COVID-19 infection.

### Risk factors.

Of the 50 enrolled patients, 88% (*n* = 36) had diabetes at the time of diagnosis. Other comorbidities noted included hypertension (30%, *n* = 15), coronary artery disease (4%, *n* = 2), hepatitis C (2%, *n* = 1), and chronic kidney disease (2%, *n* = 1). One patient had HIV/AIDS with COVID-19–associated mucormycosis. Only one patient had diabetic ketoacidosis in our series. History of steroid use and oxygen supplementation for the treatment of COVID-19 was elicited in 52% of patients (*n* = 26) and 42% of patients (*n* = 21), respectively. The duration and dosage of steroid intake varied from patient to patient, depending on physician preference.

### Clinical features.

**Figure 1.**
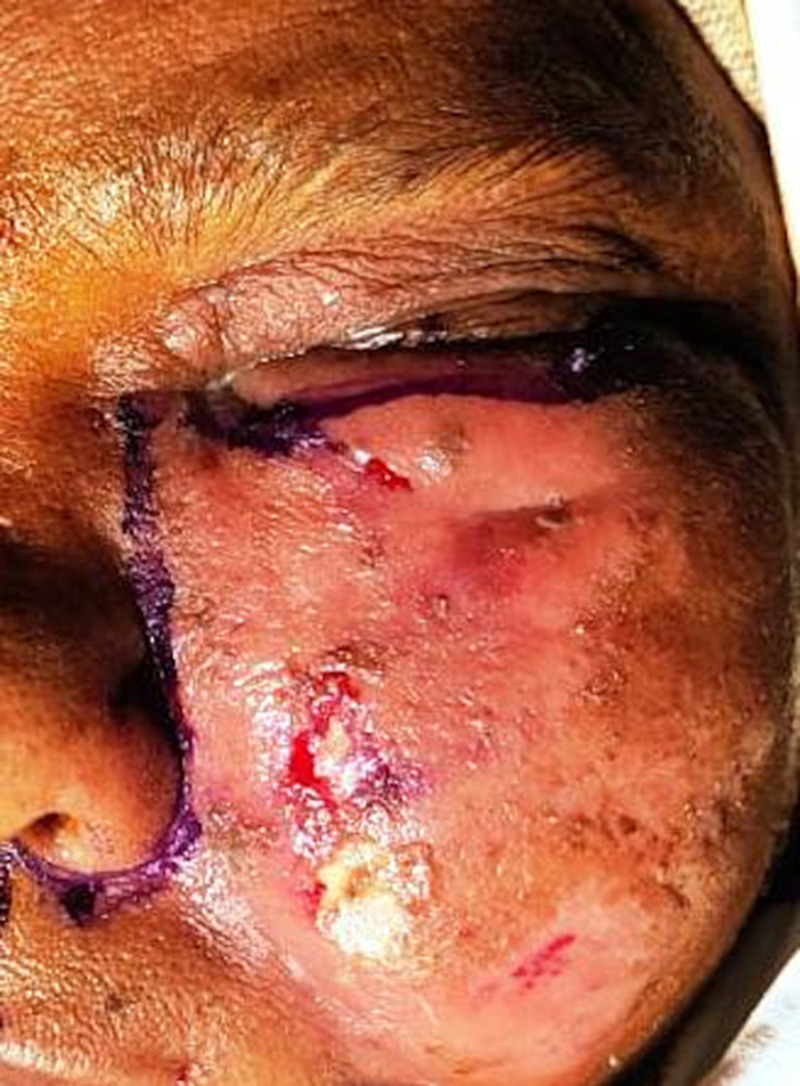
Clinical photograph showing periorbital swelling extending to the facial subcutaneous tissues in a case of COVID-19–associated sinonasal mucormycosis.

**Figure 2.**
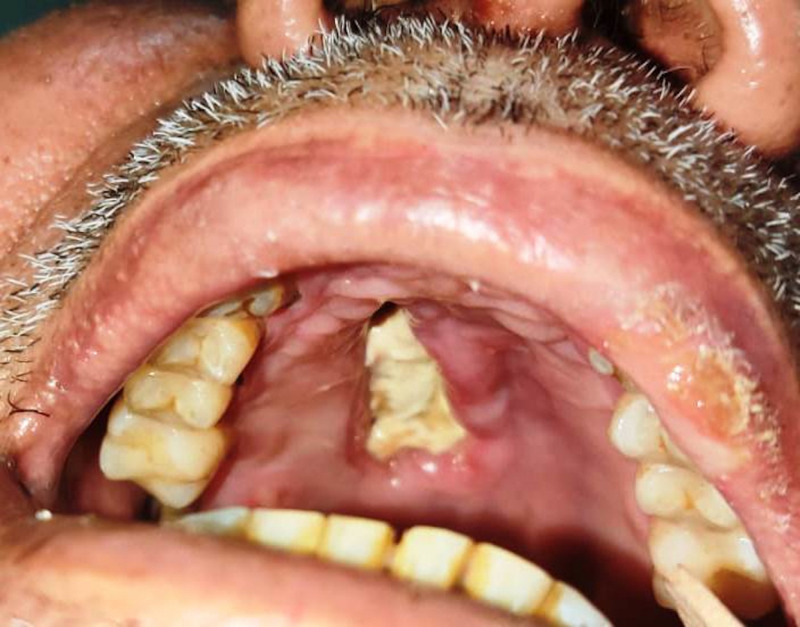
Clinical photograph depicting ulceration of the hard palate in a patient who had nasal congestion and ocular pain a few weeks after testing positive for COVID-19.

Most of the patients (84%, *n* = 42) presented to the hospital within 2 weeks of developing symptoms, with an average of 6.78 days (±3.38 days) from symptom onset. Of these patients, 35.7% (15 of 42) had concurrent COVID-19 disease—that is, they were diagnosed within the past 14 days—whereas the remaining 64.3% (*n* = 27) were post-COVID-19 status. Clinically, unilateral involvement was the norm, with only three patients with bilateral disease. Vision loss was seen in 46% of patients (*n* = 23). Headache, pain, and tenderness were the most common symptoms (40%, *n* = 20). Periorbital swelling was seen in 40% of patients (*n* = 20) (Figure 1). Other common findings were facial swelling (30*%, n* = 15) and restriction of globe movement (28%, *n* = 14) resulting from extraocular muscle involvement. Hard palate ulceration was reported in three patients (Figure 2).

### Imaging features.

#### Nasal cavity.

On imaging, a deviated nasal septum was seen in 84% of patients (*n* = 42). It was deviated toward the right in 21 patients and toward the left in 21 patients. Turbinate thickening was seen in 90% of patients (*n* = 45).

#### Paranasal sinuses.

**Figure 3.**
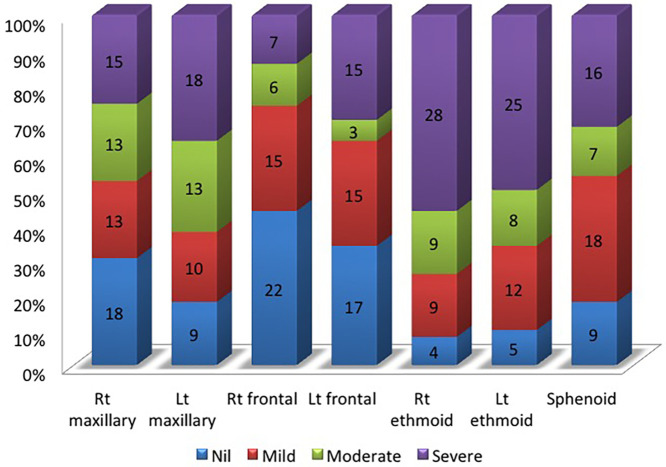
Status and degree of paranasal sinus involvement in COVID-19–associated rhino-orbital mucormycosis. Lt = left; Rt = right.

**Figure 4.**
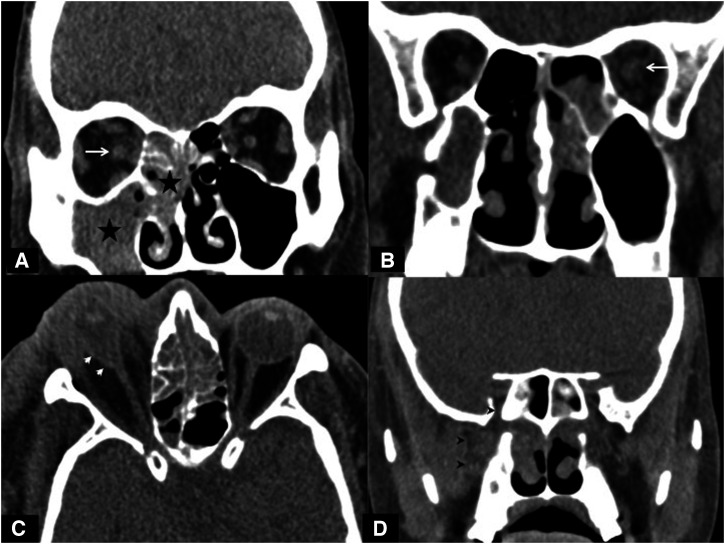
Noncontrast coronal (**A**, **B**, **D**) and axial (**C**) computed tomographic sections in different patients showing the spectrum of imaging findings in mucormycosis. Noncontrast coronal section at the level of the maxillary sinus shows severe sinusitis (asterisk, **A**) involving the maxillary and ethmoid sinuses. Fat stranding is seen in the adjacent right orbit involving the retro-orbital fat. The optic nerve is bulkier compared with the left side, with significant perioptic fat stranding (arrow, **A**). The margins of the inferior and middle recti muscles are also blurred. The optic nerve may, in addition, show loss of normal contour and ill-defined margins (arrow, **B**). The retro maxillary fat also shows fat stranding, which may extend into the pterygopalatine fossa (arrowheads, **D**). In severe orbital involvement, proptosis, thinning, and elongation of the optic nerve; loss of globe contour (arrowheads, **C**), retro-orbital fat stranding; and thickening of the overlying soft tissue suggesting orbital compartment syndrome may be seen.

The ethmoid sinuses were involved most commonly, right (92%, *n* = 46) more than left (90%, *n* = 45), followed by the maxillary, sphenoid, and frontal sinuses. Severe disease was seen more often in the ethmoid sinuses than the rest of the sinuses (Figure 3). Bilateral involvement of the ethmoid, maxillary, and frontal sinuses was seen in 82% of patients (*n* = 41), 62% of patients (*n* = 31), and 40% of patients (*n* = 20), respectively. The presence of moderate to severe sinusitis affecting two or more sinuses was seen in 94% of patients (*n* = 47) (Figure 4). The most common pattern was the involvement of both maxillary and ethmoid sinuses (88%, *n* = 44). Three or more sinus groups—among the maxillary, ethmoid, sphenoid, and frontal sinuses—were affected in 66% of patients (*n* = 33), suggesting the need for extensive sinonasal debridement to achieve adequate clearance of the disease.

**Figure 5.**
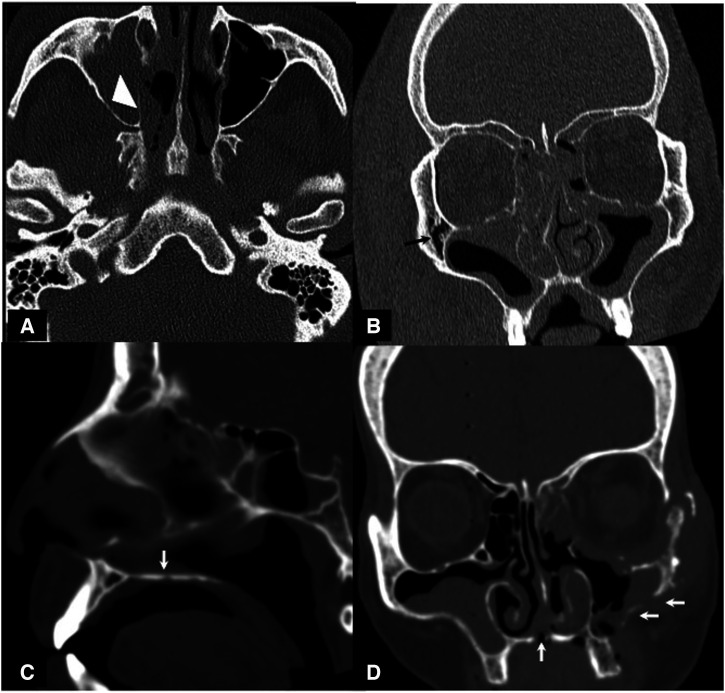
Axial (A), coronal (**B**, **D**), and sagittal (**C**) bone window sections in different patients showing the spectrum of bone involvement in mucormycosis. Most of the cases present with thinning and rarefaction of the underlying bone with extension of the disease process across the involved bone (white arrowhead, **A**). Air foci may also be seen in the bone in advanced cases (arrow, **B**). The right inferior orbital wall and bilateral lamina papyracea are also showing rarefaction in this patient. The hard palate may also show focal thinning and rarefaction (arrow, **C**). Rarely, larger defects and frank destruction may also be seen in the affected bones (arrows, **D**).

Involvement of the sinus walls in the form of multiple focal areas of bone thinning, dehiscence, and destruction was commonly seen. The right and left maxillary sinus walls were affected most often. The walls of the right and left maxillary sinuses were involved in 58% of patients (*n* = 29) and 56% of patients (*n* = 28), respectively. Involvement of the posterior wall alone was the most common finding in the afflicted maxillary sinuses [right, 86.2% (*n* = 25 of 29); left, 82.1% (*n* = 23 of 28)] followed by the erosion/destruction of the medial walls [right, 58.6% (*n* = 17 of 29); left, 53.5% (*n* = 15 of 28)]. Involvement of both the posterior and medial walls was seen in 44.8% of patients (*n* = 13 of 29) on the right side and 28.5% of patients (*n* = 8 of 28) on the left side. Bony erosion/destruction of the maxillary sinus walls was seen in 10 of 23 patients with mild disease, 20 of 26 patients with moderate disease, and 26 of 33 patients with severe disease (Figure 5). Thinning and destruction of the walls of the frontal sinuses were seen in only 4% of patients (*n* = 2 of 50) on each side, in patients with severe (*n* = 3 of 100) or moderate (*n* = 1 of 100) sinusitis. Destruction of the sphenoid sinus walls was seen in 16% of patients (*n* = 8 of 50). Of these, 75% (*n *= 6 of 8) had severe disease, and one each had moderate and mild sinusitis. In such cases, the lateral walls were more likely to be affected (*n* = 5 of 8).

Thinning and erosion of the hard palate were noted in 18% of patients (*n* = 9 of 50) (Figure 5). Cribriform plates were normal in 30% of patients (*n* = 15 of 50). Right, left, and bilateral involvement was seen in 28% of patients (*n* = 14 of 50), 12% of patients (*n* = 6 of 50), and 30% of patients (*n* = 15 of 50). Thinning and dehiscence of the lamina papyracea (Figure 4) were seen in 34% of patients (*n* = 17 of 50; right, *n* = 6 of 50; left, *n* = 10 of 50; bilateral, *n* = 1 of 50). Retromaxillary fat stranding was noted in 68% of patients (*n* = 34 of 50), indicating and directing a critical retromaxillary inspection during debridement surgery. Unilateral left retromaxillary involvement was seen in 16 of 34 patients, with right-side involvement in 17 of 34 patients. One patient had bilateral involvement. The pterygopalatine fossa was involved in 74% of patients (*n* = 37 of 50). Nineteen of 37 patients had right-side involvement; the rest (*n* = 18 of 37) had left-side involvement. Bilateral pterygopalatine fossa involvement was seen in one patient only. Severe ethmoid sinusitis was found to be associated significantly with ipsilateral pterygopalatine fossa involvement (*P* < 0.001 for both the right and left sides). Fat stranding involving the premalar and the nasolabial fat was seen in 66% of patients (*n* = 33 of 50), with right-side involvement in 16 of 33 patients and left side involvement in 17 of 33 patients. Premalar fat had normal density in 34% of patients (*n* = 17 of 50).

#### Orbits.

The orbits were also affected in multiple patients (Table 1). The extraocular muscles were involved in 64% of patients (*n* = 32 of 50). The medial recti (*n* = 23 of 32), followed by the inferior recti (*n* = 22 of 32), were most commonly involved. Simultaneous involvement of multiple extraocular muscles was common in this subgroup. Three or more extraocular muscles were affected in 18 of 32 patients. Clinically, 28% of patients (*n* = 14 of 50) had restriction of extraocular muscle movement. Of these, 9 of 14 patients displayed involvement of three or more recti on imaging. Orbital fat stranding was seen in 84% of patients (*n* = 42 of 50), indicating an orbital inspection of the periorbital and intraorbital fat during debridement (Figure 3). Proptosis of the affected eye was seen in 66% of patients (*n* = 33 of 50). Optic nerve involvement was noted in 26 of 50 patients. Irregularity of globe contour was seen in six patients only.

**Table 1:** Orbital involvement in COVID-19 associated rhino-orbital mucormycosis

Orbital involvement	Right, *n* (%)	Left, *n* (%)
Extraocular muscle involvement		
Yes	32 (64)	
No	18 (36)	
Inferior rectus	11/32 (34.37)	11/32 (34.37)
Superior rectus	10/32 (31.25)	10/32 (31.25)
Medial rectus	10/32 (31.25)	13/32 (40.62)
Lateral rectus	5/32 (15.62)	10/32 (31.25)
Superior oblique	6/32 (18.75)	8/32 (25)
Inferior oblique	0/32 (0)	2/32 (6.25)
None	–	–
Orbital compartment syndrome		
Yes	3 (6)	3 (6)
No	44 (88)	
Orbital fat stranding		
Yes	22 (44)	20 (40)
No	8 (16)	
Irregular globe contour		
Yes	3 (6)	3 (6)
No	44 (88)	
Proptosis		
Yes	17 (34)	16 (32)
No	17 (34)	
Optic nerve involvement		
Yes	13 (26)	12 (24)
Bilateral	1 (2)	
No	24 (48)	

#### Intracranial disease.

**Figure 6.**
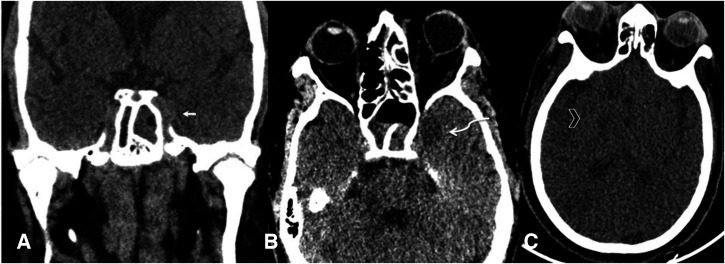
Coronal (A) and axial (**B**, **C**) computed tomographic sections showing intracranial manifestations of mucor. Cavernous sinus involvement presents as bulky cavernous sinus with loss of normal contour (arrow, **A**). The adjacent temporal lobe in the same patient also shows ill-defined hypodensity, suggesting cerebritis/abscess formation (curved arrow, **B**). Axial noncontrast computed tomography in a different patient (**C**) shows hypodensity in the right basal ganglia (arrowhead), the internal capsule, and the temporo-occipital region, suggesting infarcts.

The cavernous sinuses were seen to be affected in 10% of patients (*n* = 5 of 50) (Figure 6). Of these five patients, three had abnormal density in the ipsilateral temporal lobe as well. Brain infarcts were seen in 10% of patients (*n* = 5 of 50) on the available NCCT sections (Figure 6).

### Treatment.

Debridement was performed in 72% of patients (*n* = 36 of 50). Maxillectomy and orbital exenteration were performed in 44% of patients (*n* = 22 of 50) and 40% of patients (*n* = 20 of 50) patients. Seven patients died during their hospital stay. The remaining 43 were discharged on medical management. No symptomatic deterioration was reported until the last telephone follow-up (mean, 6 months).

## DISCUSSION

### Epidemiology.

Mucormycosis is more common in India than in the developed world, with a prevalence of around 0.14 per 1,000 population.^5^ An evaluation of the prevalence of mucormycosis at a single center in India by Prakash and Chakrabarti^10^ showed that there has been an increase in the annual incidence of cases—from 12.9 cases per year in 1990–1999 to 89 cases per year in 2013–2015. The burden of this disease has spiked in the recent second wave of COVID-19 infections in the country, as is evident in our single-center study in which 50 patients with the infection were seen in a relatively short span of time. Another study^11^ from Tamil Nadu (a southern Indian state) reported 9.5 cases per year from 2015–2019. A recent multicenter study^12^ reported 187 cases of COVID-19 associated mucormycosis from September to December 2020. The prevalence of mucormycosis among hospitalized COVID-19 patients was pegged at 0.27%, with a doubling of mucormycosis cases compared with the same in the previous year.^12^ The average age of our cohort was 54 years, with a preponderance of men, who comprised 74% of the study subjects. This is consistent with previously published series.^3^^,^^13^ Males are at a greater risk of developing severe COVID-19 infections. The chance of outdoor exposure to mucor, which is endemic in India, is also higher in the male gender.

### Risk factors.

Diabetes is the most critical risk factor for mucormycosis in India. It has been reported in 54% to 76% of patients with mucormycosis.^5^ Diabetes is also a significant risk factor for the development of ROCM. A recent multicenter study from India by Patel et al.^12^ has flagged unchecked diabetes mellitus as the most common risk factor for all clinical subtypes of mucormycosis except for renal and cutaneous mucormycosis. Diabetes in the setting of COVID-19 infection has spurred the surge of mucor infections in India. In a recent series, of the 80 cases of COVID-19 associated mucormycosis reported, 42 were from India; 8 were from the United States; 5 were from Pakistan; 4 each were from France, Iran, and Mexico; and 2 were from Russia. Single cases were reported from Austria, Bangladesh, Italy, Kuwait, Lebanon, and the United Kingdom.^14^^–^^28^

Various series^13^^,^^29^ have reported that diabetes was a contributing factor in 80% to 93% of their cases. Similarly, in our series, 88% of the patients had diabetes at the time of infection. The percentage of patients who required external oxygenation during COVID-19 treatment in our series was less than that reported by Sen et al.^13^ (41% versus 57%). History of steroid usage was also noted in only 51% of our patients, which is less than previously published data. Sen et al.^13^ mentioned that 87% of their patients had received systemic corticosteroids. Similarly, a systematic review of cases by Singh et al.^4^ revealed a history of steroid use in 76.3% of COVID-19–associated mucormycosis cases.

### Imaging.

Imaging is essential for the management of COVID-19–associated mucormycosis. It helps in diagnosing and delineating the extent of the disease. Acute, invasive rhino-orbital mucormycosis, as the name suggests, is characterized by the involvement of both the paranasal sinuses and the orbits. Thickening of the turbinates is seen in the nasal cavity.

#### Paranasal sinuses.

Paranasal sinus involvement manifests as hypodense mucosal thickening of the sinus mucosa. In 2020 in western India, Joshi et al.^2^ evaluated 25 patients with COVID-19–associated mucormycosis. Maxillary involvement was seen most commonly in their case series, followed by the ethmoid sinus.^2^ Similarly, in another series in 2021 in eastern India, Dubey et al.^30^ noted the maxillary and ethmoid sinuses were affected most frequently.^30^ The ethmoid and maxillary sinuses were involved most often in our series as well. In addition, bilateral disease and involvement of multiple sinuses were also frequently seen. Sixty-six percent of our patients had moderate to severe involvement of three or more sinus groups. This becomes significant in terms of the extent of debridement, and emphasizes the fact that patients with COVID-19, viral pneumonia, or who have an altered immune status for any reason should be warned about the warning signs and symptoms, so that an early endoscopic examination can be performed. The results of the endoscopy will affect morbidity and functional loss positively by guiding debridement. The combination of maxillary, ethmoid, frontal, and sphenoid sinusitis was noted in 26% of our patients. Mucosal thickening of the turbinates was another common finding.

#### Bony involvement.

Erosion of the sinus walls was reported by Joshi et al.^2^ in 80% of cases. Air foci within bony structures were also noted in 11 of their patients. Only one patient in their series had hard palate involvement.^2^ Dubey et al.^30^ mentioned orbital wall invasion in 60% of their patients in their series. Another series by Desai et al.,^31^ conducted in western India in 2021, reported hard palate and skull base involvement in 30% and 16% of patients, respectively. Our study did not see gross destruction of the bony margins of the paranasal sinuses and the orbits. However, multiple small foci of rarefaction and destruction of the involved bone with the extension of the disease process into the adjacent fat were noted, especially in the infratemporal fossa. Foci of erosion/destruction were seen in the walls of all sinuses in our series, including the maxillary, ethmoidal, frontal, and sphenoidal sinuses. The posterior maxillary sinus walls were involved most commonly. This finding was crucial in surgical decision making and resulted in the exploration of retromaxillary fat by removing the posterior wall of maxillary sinus in affected patients. The retromaxillary, infratemporal area and the pterygoid bones were debrided if found to be necrotic. Without CT, the retromaxillary area (being a hidden area for both clinical and intraoperative examination) may be overlooked and may escape disease clearance. The singular principle of mucormycosis debridement surgery—“debride” til it bleeds—would then be defeated. Although most of the patients with bone destruction or erosion of the maxillary walls had moderate to severe sinusitis, 43% of patients with mild maxillary sinus disease also had imaging features consistent with bone involvement, suggesting that mild maxillary sinusitis does not preclude the extension of the disease to the bone. Thus, it is essential to evaluate the bony structures carefully in all cases. Hard palate involvement was noted in 18% of our patients, which has significant concerns in terms of postoperative swallowing function and rehabilitation.

#### Locoregional extension.

Extension of the disease process into surrounding soft tissue was also a common finding in our series. The infratemporal region and the premalar/nasolabial fat were involved in 68% and 66% of our patients, respectively. Hence, a significant percentage (> 60%) of our patients had loss of facial skin, resulting in significant cosmetic deformities and concerns after debridement and medical management, which affected quality of life and required additional posttreatment reconstructive surgeries 6 months after being disease free. These findings are consistent with the data published by Dubey et al.^30^

#### Orbital involvement.

Spread of infection to the orbit may occur as a result of direct spread across the lamina papyracea or via vascular/perineural channels.^31^ Orbital fat stranding and involvement of the recti and optic nerves were seen. Orbital fat stranding was identified easily on NCCT and was seen in 84% of our patients. It was significantly greater when compared with other observational studies in which the prevalence ranged from 40% to 70%.^13^^,^^30^^,^^31^ This may be a result of selection bias, as our institute is a tertiary care hospital, where only advanced cases were being referred. Clinicians and surgeons dealing with management should be aware and warned of the finding of orbital fat stranding, and should look for the same in the CT scans of each and every suspected case of mucormycosis. Extraocular muscle involvement was seen in 64% of our patients. The medial and inferior recti were involved most often. Similar findings were also reported by Dubey et al.^30^

### Isolated mucormycosis versus COVID-19–associated mucormycosis.

Imaging in non-COVID-19 mucormycosis is similar to COVID-19–related mucormycosis. Son et al.^32^ evaluated 14 patients with mucormycosis. They reported mucosal thickening of the involved paranasal sinuses, with complete opacification and air–fluid levels in patients with rhino-orbital mucormycosis. They also reported early involvement of the extraocular muscles in rhino-orbital mucor compared with bacterial orbital cellulitis. Imaging features in non-COVID-19 mucor were also studied by Therakathu et al.^33^ in 34 patients. The ethmoid sinuses were involved most commonly, with multiple sinus involvement, in their series. Unilateral involvement was more common than bilateral. They also described extension into facial, retromaxillary, and orbital fat. Sixty percent of their patients had normal walls spread across uninvolved walls. The nasal cavity revealed nonspecific turbinate hypertrophy. The most common sites of extrasinus extension were the orbits and the face. Other areas of spread included the pterygopalatine fossa, skull base, cavernous sinuses, and brain.^16^ This pattern of involvement is similar to our findings.

### Surgical aspects.

An interesting observation in our series was the number of patients with orbital involvement as evidenced in CT scans (64%, *n* = 32) was less than the number of patients presenting with proptosis (66%, *n* = 33) and greater than the number of patients presenting with visual deterioration (66%, *n* = 23). This was again greater than the number of patients requiring orbital exenteration (40%, *n* = 20), which was a decision made intraoperatively based on the viability/involvement of the tissues (such as a breach in the periorbita, and disease causing necrosis or change of color of the orbital fat). Three of our patients developed vision deterioration despite having normal orbital fat and periorbita. Similarly, there were many clinically subtle cases with CT signs of orbital involvement without frank intraorbital necrosis, suggesting vascular channels of involvement. The greater percentage of bony erosion and foci of destruction in our series may be attributed to the submillimeter resolution provided by the current generation of 128-slice CT scanners. This emphasizes the fact that orbital tissue inspection by incising the periorbita should be an integral part of debridement surgery performed for mucormycosis. This would ensure complete disease clearance. Moreover, it would also help in assessing the orbital extent of the infection and whether the eyeball may be saved.

Our study had certain limitations. First, the sample size was limited and may not have been sufficient to derive an accurate radiological–clinical correlation. Second, because only NCCT was performed as a frontline investigation in all patients, the adequacy of clinical interpretation for evaluating the disease and its extent may be limited. However, we believe that the significant findings identified in our evaluation offset all such limitations.

## CONCLUSION

COVID-19–associated ROCM is an acute, invasive fungal disease with high morbidity and mortality. Our study shows typical bony involvement, with locoregional spread identified in the form of multiple small areas of erosion and focal areas of destruction, leaving the rest of the bone intact. A spread across bony structures without frank destruction suggests a vascular route for the spread, in addition to direct extension. Bilaterality of the disease was seen more often in our cohort compared with non-COVID mucormycosis, in which unilateral disease is more prevalent. In an appropriate clinical setting, NCCT serves well in aiding diagnosis and evaluating disease extension, thereby proving decisive in the majority of patients. This also highlights the need for having a low threshold for exploration through the posterior wall of the maxillary sinus with the slightest suggestion of retromaxillary involvement. MRI can be reserved as a problem-solving tool in complex cases. Moreover, there may be sterilization issues in using MRI in COVID-19 patients in centers where only a standalone MR scanner is available. Noncontrast CT may thus be used as a primary modality for managing ROCM, especially during emergency conditions such as the COVID pandemic, where excessive patient load was seen over an extremely short interval of time. In addition, this approach reduces unnecessary MRI examinations, thereby reducing exposure of health-care workers to infectious agents (COVID-19 in the current scenario). Our study also shows that there is no remarkable difference in the imaging findings between COVID-19 and non-COVID-19 mucormycosis.

## Supplemental Materials


Supplemental materials

## References

[1] Song G Liang G Liu W, 2020. Fungal co-infections associated with global COVID-19 pandemic: a clinical and diagnostic perspective from China. Mycopathologia 31: 1–8. 10.1007/s11046-020-00462-9 PMC7394275 32737747

[2] Joshi AR Muthe MM Patankar SH Athawale A Achhapalia Y, 2021. CT and MRI findings of invasive mucormycosis in the setting of COVID-19: experience from a single center in India. AJR Am J Roentgenol 217: 1431–1432. 34161127 10.2214/AJR.21.26205

[3] Aggarwal SK Kaur U Talda D Pandey A Jaiswal S Kanakan A Singh A Chakrabarti SS, 2021. Case report: rhino-orbital mucormycosis related to COVID-19: a case series exploring risk factors. Am J Trop Med Hyg 106: 566–570. 34902834 10.4269/ajtmh.21-0777 PMC8832906

[4] Singh AK Singh R Joshi SR Misra A, 2021. Mucormycosis in COVID-19: a systematic review of cases reported worldwide and in India. Diabetes Metab Syndr 15: 102146. 34192610 10.1016/j.dsx.2021.05.019 PMC8137376

[5] Prakash H Chakrabarti A, 2021. Epidemiology of mucormycosis in India. Microorganisms 9: 523. 33806386 10.3390/microorganisms9030523 PMC8000977

[6] Caetano LA Faria T Springer J Loeffler J Viegas C, 2019. Antifungal-resistant Mucorales in different indoor environments. Mycology 10: 75–83. 31069121 10.1080/21501203.2018.1551251 PMC6493325

[7] Nicolás FE Murcia L Navarro E Navarro-Mendoza MI Pérez-Arques C Garre V, 2020. Mucorales species and macrophages. J Fungi (Basel) 6: 94. 32604972 10.3390/jof6020094 PMC7344864

[8] Prakash H Ghosh AK Rudramurthy SM Paul RA Gupta S Negi V Chakrabarti A, 2016. The environmental source of emerging *Apophysomyces variabilis* infection in India. Med Mycol 54: 567–575. 27118802 10.1093/mmy/myw014

[9] Vaughan C Bartolo A Vallabh N Leong SC, 2018. A meta-analysis of survival factors in rhino-orbital-cerebral mucormycosis: has anything changed in the past 20 years? Clin Otolaryngol 43: 1454–1464. 29947167 10.1111/coa.13175

[10] Prakash H Chakrabarti A, 2021. Epidemiology of mucormycosis in India. Microorganisms 9: 523. 33806386 10.3390/microorganisms9030523 PMC8000977

[11] Priya P Ganesan V Rajendran T Geni VG, 2020. Mucormycosis in a tertiary care center in South India: a 4-year experience. Indian J Crit Care Med 24: 168–171. 32435094 10.5005/jp-journals-10071-23387 PMC7225759

[12] Patel A , 2021. Multicenter epidemiologic study of coronavirus disease–associated mucormycosis, India. Emerg Infect Dis 27: 2349–2359. 34087089 10.3201/eid2709.210934 PMC8386807

[13] Sen M , 2021. Epidemiology, clinical profile, management, and outcome of COVID-19-associated rhino-orbital-cerebral mucormycosis in 2826 patients in India: collaborative OPAI-IJO study on mucormycosis in COVID-19 (COSMIC), report 1. Indian J Ophthalmol 69: 1670–1692. 34156034 10.4103/ijo.IJO_1565_21 PMC8374756

[14] Hoenigl M , 2022. The emergence of COVID-19 associated mucormycosis: a review of cases from 18 countries. Lancet Microbe 3: e543–e552. 35098179 10.1016/S2666-5247(21)00237-8 PMC8789240

[15] Zurl C Hoenigl M Schulz E Hatzl S Gorkiewicz G Krause R Eller P Prattes J, 2021. Autopsy proven pulmonary mucormycosis due to *Rhizopus microsporus* in a critically ill COVID-19 patient with underlying hematological malignancy. J Fungi (Basel) 7: 88. 33513875 10.3390/jof7020088 PMC7912223

[16] Garg D Muthu V Sehgal IS Ramachandran R Kaur H Bhalla A Puri GD Chakrabarti A Agarwal R, 2021. Coronavirus disease (Covid-19) associated mucormycosis (CAM): case report and systematic review of literature. Mycopathologia 186: 289–298. 33544266 10.1007/s11046-021-00528-2 PMC7862973

[17] Werthman-Ehrenreich A, 2021. Mucormycosis with orbital compartment syndrome in a patient with COVID-19. Am J Emerg Med 42: 264.e5–264.e8. 10.1016/j.ajem.2020.09.032 PMC7493738 32972795

[18] Rabagliati R Rodríguez N Núñez C Huete A Bravo S Garcia P, 2021. COVID-19-associated mold infection in critically ill patients, Chile. Emerg Infect Dis 27: 1454–1456. 33760726 10.3201/eid2705.204412 PMC8084475

[19] Khatri A Chang KM Berlinrut I Wallach F, 2021. Mucormycosis after coronavirus disease 2019 infection in a heart transplant recipient: case report and review of literature. J Mycol Med 31: 101125. 33857916 10.1016/j.mycmed.2021.101125 PMC8017948

[20] Sarkar S Gokhale T Choudhury SS Deb AK, 2021. COVID-19 and orbital mucormycosis. Indian J Ophthalmol 69: 1002–1004. 33727483 10.4103/ijo.IJO_3763_20 PMC8012924

[21] Khan N Gutierrez CG Martinez DV Proud KC, 2021. A case report of COVID-19 associated pulmonary mucormycosis. Arch Clin Cases. 7: 46–51. 34754927 10.22551/2020.28.0703.10172 PMC8565698

[22] Alekseyev K Didenko L Chaudhry B, 2021. Rhinocerebral mucormycosis and COVID-19 pneumonia. J Med Cases 12: 85–89. 33984095 10.14740/jmc3637 PMC8040444

[23] Karimi-Galougahi M Arastou S Haseli S, 2021. Fulminant mucormycosis complicating coronavirus disease 2019 (COVID-19). Int Forum Allergy Rhinol 11: 1029–1030. 33713565 10.1002/alr.22785 PMC8250489

[24] Waizel-Haiat S Guerrero-Paz JA Sanchez-Hurtado L Calleja-Alarcon S Romero-Gutierrez L, 2021. A case of fatal rhino-orbital mucormycosis associated with new onset diabetic ketoacidosis and COVID-19. Cureus 13: e13163. 33575155 10.7759/cureus.13163 PMC7870113

[25] Kanwar A Jordan A Olewiler S Wehberg K Cortes M Jackson BR, 2021. A fatal case of *Rhizopus azygosporus* pneumonia following COVID-19. J Fungi (Basel) 7: 174. 33670842 10.3390/jof7030174 PMC7997212

[26] Bellanger AP Navellou JC Lepiller Q Brion A Brunel AS Millon L Berceanu A, 2021. Mixed mold infection with *Aspergillus fumigatus* and *Rhizopus microsporus* in a severe acute respiratory syndrome coronavirus 2 (SARS-CoV-2) patient. Infect Dis Now 51: 633–635. 33527098 10.1016/j.idnow.2021.01.010 PMC7839422

[27] Mekonnen ZK Ashraf DC Jankowski T Grob SR Vagefi MR Kersten RC Simko JP Winn BJ, 2021. Acute invasive rhino-orbital mucormycosis in a patient with COVID-19-associated acute respiratory distress syndrome. Ophthalmic Plast Reconstr Surg 37: e40–e80. 33229953 10.1097/IOP.0000000000001889 PMC7927902

[28] Hanley B , 2020. Histopathological findings and viral tropism in UK patients with severe fatal COVID-19: a post-mortem study. Lancet Microbe 1: e245–e253. 32844161 10.1016/S2666-5247(20)30115-4 PMC7440861

[29] John TM Jacob CN Kontoyiannis DP, 2021. When uncontrolled diabetes mellitus and severe COVID-19 converge: the perfect storm for mucormycosis. J Fungi (Basel) 7: 298. 33920755 10.3390/jof7040298 PMC8071133

[30] Dubey S , 2021. COVID-19 associated rhino-orbital-cerebral mucormycosis: an observational study from eastern India, with special emphasis on neurological spectrum. Diabetes Metab Syndr 15: 102267. 34509790 10.1016/j.dsx.2021.102267 PMC8407938

[31] Desai SM Gujarathi-Saraf A Agarwal EA, 2021. Imaging findings using a combined MRI/CT protocol to identify the “entire iceberg” in post-COVID-19 mucormycosis presenting clinically as only “the tip.” Clin Radiol 76: 784.e27–784.e33. 10.1016/j.crad.2021.07.002 34353524

[32] Son JH Lim HB Lee SH Yang JW Lee SB, 2016. Early differential diagnosis of rhino-orbito-cerebral mucormycosis and bacterial orbital cellulitis: based on computed tomography findings. PLoS One 11: e0160897. 27501044 10.1371/journal.pone.0160897 PMC4976984

[33] Therakathu J Prabhu S Irodi A Sudhakar SV Yadav VK Rupa V, 2018. Imaging features of rhinocerebral mucormycosis: a study of 43 patients. Egypt J Radiol Nucl Med 49: 447–452.

