# Supplementary material for: Clinicoradiological Profile of COVID-19–Associated Rhino-Orbital Cerebral Mucormycosis with a Focus on Computed Tomography: A Clinical Case Series and Review
Source: Am J Trop Med Hyg. 2023 Jul 24;109(3):600–7. doi: 10.4269/ajtmh.22-0298 (PMC10484265; doi:10.4269/ajtmh.22-0298)
Supplement: Supplementary file 1 [file tpmd220298.SD1.pdf]

Postgraduate Institute of Medical Education and Research, Chandigarh
Institutional Ethics Committee

Prof. Samir Malhotrta
Chairman

Assoc. Prof. Mona Duggal
Convener

Chairman

Prof. Samir Malhotra

Members

Dr. G.R.V. Prasad

Dr. K. Mukhopadhyaya

Dr. Parampreet K.

Dr. Amanjit Bal

Dr. Ashish Sharma

Dr. Rijuneeta

Dr. Ramandeep Singh

Dr. Nirmal Raj

Dr. Narender Kumar

Convener

Dr. Mona Duggal

Contact Office:
+91 172 2755266

No: INT/IEC/2023/SPL-36

Date:- 07-01-2023

Dr. Chirag Kamal Ahuja,
Dept. of Radiodiagnosis,
PGIMER, Chandigarh.

Title: - **Clinicoradiological profile of Covid associated rhino-orbital mucormycosis with focus on computed Tomography: a clinical case series and review.**

Reference No: - IEC-INT/2023/Study-887

Dear Dr. Ahuja,

The Institute Ethics Committee (Intramural) PGIMER, Chandigarh was received the above reference retrospective study proposal. It was decided that this proposal is exempt from review by the Ethics committee.

In all future correspondence with respect to your project, please attach a photocopy of this letter.

With kind regards,

Yours sincerely,

Mona Duggal

(Dr. Mona Duggal)
Convener, Instt. Ethics Committee (Intramural),
PGIMER, Chandigarh.

Copy to: - Medical Education Cell, PGIMER, Chandigarh.
